# Evaluation of diagnostic methods for the detection of intestinal schistosomiasis in endemic areas with low parasite loads: Saline gradient, Helmintex, Kato-Katz and rapid urine test

**DOI:** 10.1371/journal.pntd.0006232

**Published:** 2018-02-22

**Authors:** Warllem Junio Oliveira, Fernanda do Carmo Magalhães, Andressa Mariana Saldanha Elias, Vanessa Normandio de Castro, Vivian Favero, Catieli Gobetti Lindholz, Áureo Almeida Oliveira, Fernando Sergio Barbosa, Frederico Gil, Maria Aparecida Gomes, Carlos Graeff-Teixeira, Martin Johannes Enk, Paulo Marcos Zech Coelho, Mariângela Carneiro, Deborah Aparecida Negrão-Corrêa, Stefan Michael Geiger

**Affiliations:** 1 Departamento de Parasitologia, Instituto de Ciências Biológicas, Universidade Federal de Minas Gerais, Belo Horizonte, Brasil; 2 Grupo de Parasitologia Médica, Pontifícia Universidade Católica de Rio Grande do Sul, Porto Alegre, Brasil; 3 Laboratório de Esquistossomose, Centro de Pesquisas René Rachou, Fundação Oswaldo Cruz, Belo Horizonte, Brasil; 4 Secretaria de Vigilância em Saúde, Instituto Evandro Chagas, Ministério da Saúde, Belém, Brasil; Leiden University Medical Center, NETHERLANDS

## Abstract

**Background:**

In some tropical countries, such as Brazil, schistosomiasis control programs have led to a significant reduction in the prevalence and parasite burden of endemic populations. In this setting, the Kato-Katz technique, as the standard diagnostic method for the diagnosis of *Schistosoma mansoni* infections, which involves the analysis of two slides from one fecal sample, loses its sensitivity. As a result, a significant number of infected individuals are not detected. The objective of this study was to perform extensive parasitological testing of up to three fecal samples and include a rapid urine test (POC-CCA) in a moderate prevalence area in Northern Minas Gerais, Brazil, and evaluate the performance of each test separately and in combination.

**Methods and findings:**

A total of 254 individuals were examined with variants of the standard Kato-Katz technique (up to18 Kato-Katz slides prepared from three fecal samples), a modified Helmintex (30 g of feces), the saline gradient (500 mg of feces), and the POC-CCA methods. We established a reference standard taking into consideration all the positive results in any of the parasitological exams. Evaluation of the parasite burden by two Kato-Katz slides confirmed that most of the individuals harbored a light infection. When additional slides and different parasitological methods were included, the estimated prevalence rose 2.3 times, from 20.4% to 45.9%. The best sensitivity was obtained with the Helmintex method (84%). All parasitological methods readily detected a high or moderate intensity of infection; however, all lost their high sensitivity in the case of low or very low intensity infections. The overall sensitivity of POC-CCA (64.9%) was similar to the six Kato-Katz slides from three fecal samples. However, POC-CCA showed low concordance (κ = 0.34) when compared with the reference standard.

**Conclusions:**

The recommended Kato-Katz method largely underestimated the prevalence of *S*. *mansoni* infection. Because the best performance was achieved with a modified Helmintex method, this technique might serve as a more precise reference standard. An extended number of Kato-Katz slides in combination with other parasitological methods or with POC-CCA was able to detect more than 80% of egg-positive individuals; however, the rapid urine test (POC-CCA) produced a considerable percentage of false positive results.

## Author summary

Human infection with the flatworm *Schistosoma mansoni* continues to be a public health problem in many tropical countries, including Brazil. The parasitological method recommended by the World Health Organization for the detection of intestinal schistosomiasis, the Kato-Katz method (KK), underestimates the prevalence of the infection in endemic areas with reduced parasite burden. When extensive and supplementary parasitological exams were performed, the prevalence of schistosomiasis in the examined population increased 2.3 times. Additional KK slides and other parasitological methods, such as saline gradient and Helmintex, allowed us to establish a strong reference standard that was used to assess the parasitological tests and the rapid urine test for the detection of the circulating cathodic antigen of *S*. *mansoni* (POC-CCA). All tests readily detected the presence of the flatworm in individuals with medium to high parasite loads. The Helmintex method showed the best performance as it detected almost 84% of all infected individuals. A variant of the standard KK method, involving six fecal smears from three stool samples, detected two-thirds of all infections, thus having a performance comparable to that found with the POC-CCA. A combination of this variant KK method with the POC-CCA may be a field-applicable alternative to improve the diagnosis of *S*. *mansoni* infections in individuals with low parasite loads in endemic areas.

## Introduction

Recent estimates of helminth infections indicate the existence of more than one billion infected individuals in underdeveloped areas of Africa, Asia, and in Central and South America [1]. Among the different trematode species infecting humans, schistosome species are the parasites with the highest impact on public health, affecting more than 240 million individuals and with 700–800 million people living at risk of infection [2–4]. In sub-Saharan Africa, approximately 280,000 deaths per annum have been attributed to schistosome infections and their clinical complications [5]. In Brazil, the only schistosome species transmitted among the human population is *Schistosoma mansoni* and estimates vary between 1.5 and 6 million infected individuals [1, 6, 7, 8, 9].

Since the implementation of the National Schistosomiasis Control Program (NSCP) in the 1970s and decades of consequent chemotherapeutic interventions, the Brazilian health authorities reported significant improvements in terms of transmission, prevalence, and parasite load in the country’s endemic regions, especially in the states of Minas Gerais and Bahia [10]. In this new epidemiological scenario, most of the infected individuals in endemic areas harbor low parasite loads and are very unlikely to be detected with the commonly used parasitological methods [11,12].

The Kato-Katz method (KK) [13] is recommended by the World Health Organization (WHO) as the standard method for the detection of *S*. *mansoni* infection [14–16]. It is very efficient in individuals with high to medium parasite loads, e.g. more than 100 eggs per gram of feces, but shows reduced sensitivity in individuals with low parasite loads. As a consequence, the real prevalence in an endemic setting may be significantly underestimated and that has led to shortcomings in the control of schistosomiasis in these areas [17–20]. An important result of the NSCP was a significant reduction in the number of severe clinical cases and deaths due to *S*. *mansoni* infection [21, 22]. However, the failure to correctly identify all or most of the individuals with low parasite burden by the standard parasitological approach (1 or 2 KK slides) has contributed to the continuation of *S*. *mansoni* infection, with accompanying contamination of the environment, especially the water bodies, and hence, allow reinfection in endemic areas. Therefore, if new WHO guidelines about the elimination of schistosome infections in the world are sought to be achieved [16], new and more sensitive methods, apart from the standard KK test, will have to be applied.

Due to the reduced performance of the KK method for the diagnosis of *S*. *mansoni* infection in areas with low endemicity, new parasitological methods have been developed such as saline gradient [23] and Helmintex [24]. Even immunological methods have been re-evaluated in order to improve detection of *S*. *mansoni* infection in endemic populations [25–27]. As an alternative to enhance the specificity of immunological methods for the diagnosis of schistosome infections, some assays focus on the detection of parasite-secreted antigens in serum or urine samples of infected individuals [28]. Indeed, circulating cathodic antigens (CCA) of *S*. *mansoni* are released into the circulation by juvenile and adult schistosomes and the levels of these antigens correlate with the worm burden, thus indicating active infection [29–31]. Based on these initial studies, a rapid antigen test, the Point-of-Care-CCA rapid test (POC-CCA) was developed and is commercially available. It detects the circulating antigen in urine samples and has a higher sensibility than the standard KK method when it was evaluated in schistosomiasis endemic areas in Africa [32–35]. However, most of these studies were restricted to Africa and they only compared the POC-CCA reactivity in urine samples with parasitological results obtained with the standard KK method and using this method as the reference standard [36]. Since the KK method is not sensitive enough to identify individuals with low parasite burden and serve as a ‘gold standard’, the real efficiency of the POC-CCA to detect *S*. *mansoni* infection in endemic populations remains to be validated in relation to more sensitive parasitological, molecular, and serological methods.

In the present study, we performed a combination of alternative parasitological methods to detect more precisely intestinal schistosomiasis in an endemic area in Brazil. The thorough parasitological investigation allowed us to implement a new reference standard to detect active *S*. *mansoni* infection and to evaluate each of the parasitological methods for its performance and accuracy. Moreover, we analyzed the potential of POC-CCA rapid urine test as an alternative for time-consuming parasitological exams in detecting individuals with low parasite burden commonly found in endemic areas subjected to long-term chemotherapeutic interventions.

## Materials and methods

### Ethics statement

The present study was approved by the Ethics Committee of the Research Center René Rachou—FIOCRUZ and all project details have been registered on the Brazilian Platform for Research with Human Subjects (Plataforma Brasil) under the following number: CAAE#21824513.9.0000.5091. Before any research activities, the local health authorities were contacted and agreed to collaborate with the researchers from the different institutions. All enrolled participants were required to sign an informed consent form. Parents or legal guardians signed the informed consent when minors were involved.

When the parasitological results were positive, the relevant individuals were informed and received free oral treatment at the local health clinic. Schistosomiasis: praziquantel (40 mg/kg for adults and 60 mg/kg for children); intestinal helminths: albendazole (400 mg); protozoan parasites: metronidazole (250 mg/2x/ 5 days).

### Study area and population

The study was conducted in a rural area of the district of Brejo do Amparo, Municipality of Januária (S1 Fig Supplemental Information), located in the northern part of Minas Gerais State, Brazil, approximately 600 km from the capital Belo Horizonte. The community is located along the margins of the Tocantins brook and consists of roughly 270 individuals in total. In local meetings and house-to-house visits, the project was explained to all interested inhabitants, and stool exams were offered. A family-based socio-economic questionnaire was applied to gather information on household construction, water supply, sanitation, and other socio-economical aspects. Also, an individual questionnaire was used to record demographic and occupational information and to indicate previous clinical conditions that might be relevant for the research. Based on past interventions carried out by the local health authorities responsible for schistosomiasis control, a prevalence of *S*. *mansoni* infection between 15–20% was expected in this area. According to these authorities, no schistosomiasis control interventions had been performed in the localities during the last two years before the beginning of the present study.

### Collection of biological samples and laboratory procedures

**Fig 1:**
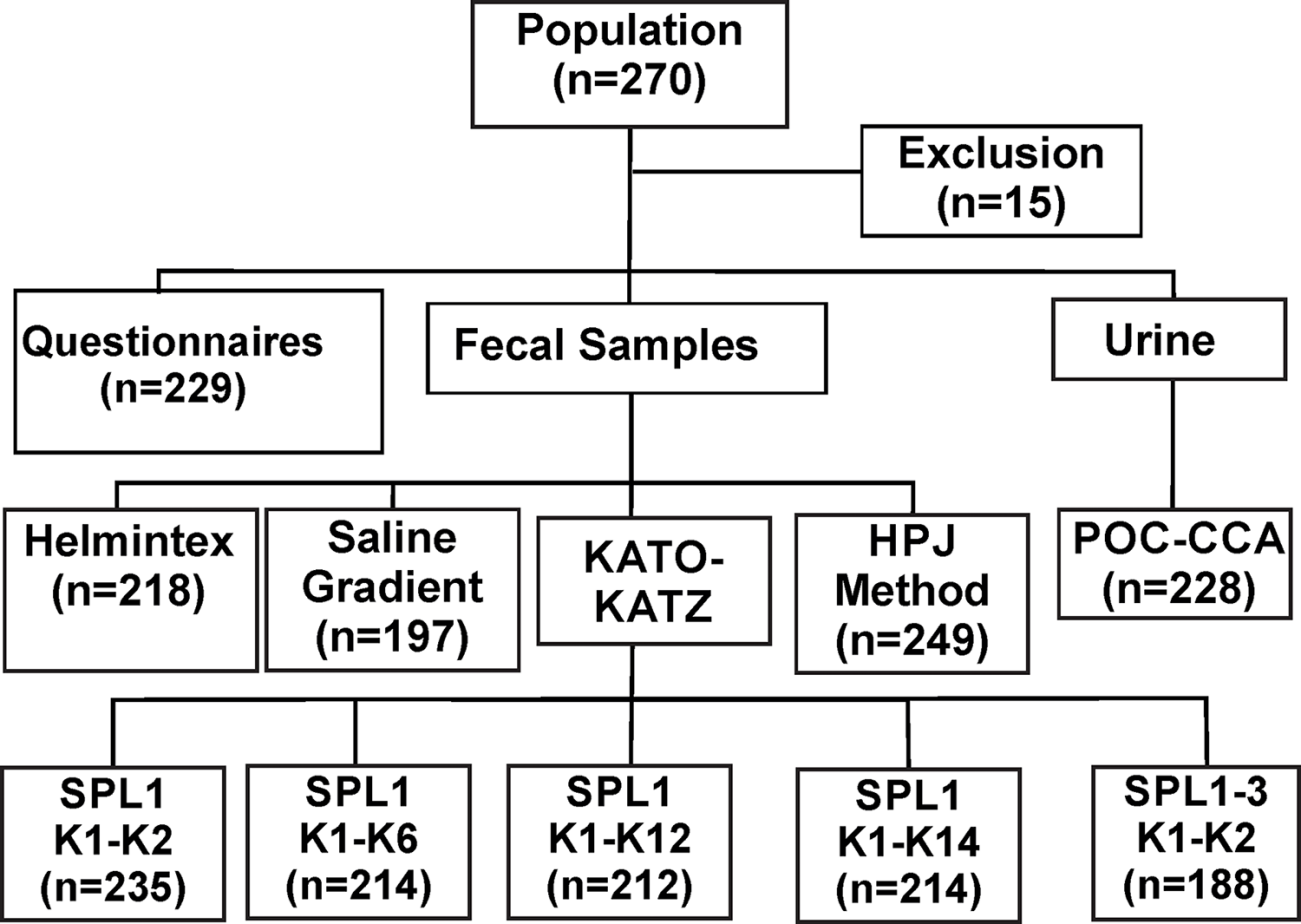
Flowchart describing the workflow for the diagnosis of intestinal schistosomiasis in an endemic population within the district of Brejo do Amparo, Januária, Minas Gerais, Brazil. Fecal samples were examined with the Kato-Katz technique with one fecal sample and two (SPL1 K1-K2), six (SPL1 K1-K6), 12 (SPL1 K1-K12), and 14 thick-smears (SPL1 K1-K14), or with three fecal samples with two slides each (SPL1-3 K1-K2), saline gradient, Helmintex and spontaneous sedimentation technique (HPJ). Further, individual urine samples were analyzed with the point-of-care rapid urine test (POC-CCA) that detects the circulating cathodic antigen of *Schistosoma mansoni*. The numbers in brackets indicate the number of individuals tested with each method.

Participants were asked to provide a urine sample and three fecal samples, which were collected on consecutive days. Fecal samples were brought to the field laboratory in Januária to be processed by the different parasitological methods. The flow diagram in Fig 1 shows the total number of samples analyzed by each parasitological test and the results obtained with the rapid urine test (POC-CCA). At least 50 grams of feces were collected with the first fecal sample using a 500 ml plastic container, which is sufficient for a complete fecal evacuation. The fecal samples collected in the following days were small and, therefore, 80 ml plastic cups were used.

Variants of the standard KK technique [13] were perfomed by preparing 14 slides with the first fecal sample and two slides for the second, and third samples. Slides were examined under the microscope (100x) for the presence of *S*. *mansoni* eggs and other intestinal helminths. The exams were conducted by experienced microscopists at the Centro de Pesquisas René Rachou and the Universidade Federal de Minas Gerais. At least 15% of all slides had their reading confirmed by a second microscopist, after random selection. The intensity of infection was calculated by determining the mean number of *S*. *mansoni* eggs found in each slide and multiplying the mean obtained by 24 to determine the number of eggs per gram of feces (EPG). According to the World Health Organization [14], the intensity of *S*. *mansoni* infection can be categorized as light (1–99 EPG), moderate (100–399 EPG), or heavy (≥400 EPG). The spontaneous sedimentation method [37] was used to evaluate the presence of protozoan parasites in fecal samples.

Next, a subsample was taken from the first fecal sample and processed following the saline gradient technique and a modified Helmintex method. For the saline gradient method [23], a suspension of 500 mg of feces was subjected to a slow flow of a 3% saline solution during one hour. Subsequently, the supernatant was removed and the sediment was placed onto microscope slides to search for S. *mansoni* eggs. The modified Helmintex method was performed as described by Favero and colleagues [38]. Briefly, 30 grams of feces from the first fecal sample were suspended in 70% ethanol, treated with detergent (Tween-20), subjected to repetitive filtration and sedimentation steps, the addition of a solution with magnetic particles, and the separation of *S*. *mansoni* eggs using a magnetic field. Finally, the free suspension was discarded and the attached particles, which formed the final sediment, were mixed with 3% ninhydrin solution and transferred onto microscope slides to search for *S*. *mansoni* eggs [38].

As mentioned above, each participant was also asked to provide a urine sample to perform the rapid urine test (POC-CCA, Rapid Medical Diagnostics, Pretoria, South Africa) and detect the circulating cathodic antigen of *S*. *mansoni*. To this end, first-morning urine samples were collected, transferred to the field laboratory in Januária, aliquoted in 10–15 ml samples, and stored at -20°C until further testing. The test followed the manufacturers’ guidelines, and was read 20 minutes after addition of the urine sample and buffer solution. Test results were scored as negative if the circulating cathodic antigen band was absent. Positive results were scored as trace (very light band), weak (+), medium (++) and strong (+++) depending on the intensity of the circulating cathodic antigen band [28, 39]. Cases with trace results for the circulating cathodic antigen of *S*. *mansoni* were considered as positive. The tests were scored independently by two investigators. In case of conflicting results, a third investigator was consulted.

### Statistical analyses and performance of the parasitological methods

Analyses were performed using Open Epi, version 3.03 and GraphPad Prism, version 5.0. In order to evaluate the performance of the different diagnostic tests, a “Reference Standard” was established, which included all positive results (visible eggs) from any of the parasitological methods used (18 KK slides, saline gradient, and Helmintex). Normal distribution of the data was verified by the Shapiro-Wilk test. For non-parametric data and categorical variables, the Chi-square test was used. To compare the means for continuous variables, the Manny-Whitney U-test and Kruskal-Wallis test were used, with a *p*-value ≤ 0.05 considered significant. The overall prevalence of *S*. *mansoni* infection in the endemic area was calculated by the number of egg-positive individuals found in any of the parasitological exams, as defined by the “Reference Standard”, divided by the total number of participants. To compare the performance and accuracy of each method, we calculated the sensitivity, specificity, positive (PPV) and negative predictive values (NPV), and concordance (kappa index). To evaluate the degree of concordance between the different methods, the kappa index (κ), which varies from 0 to 1.0, followed the following categorization: no agreement if κ<0.01; bad if 0.01≤κ≤0.20; weak if 0.21≤κ≤ 0.40; moderate if 0.41≤κ≤0.60; good if 0.61≤κ≤0.80, and excellent agreement if κ>0.81 [40]. The relationship between the intensity of infection, as determined by the mean EPG value of two slides from the first fecal sample and the semi-quantitative intensity of POC-CCA results was examined by the Spearman’s rank correlation test.

## Results

### Characterization of the study population

As shown in Table 1, the parasitological study included 257 individuals, of which 122 were male (47.5%) and 135 female (52.5%). Age of the participants ranged from 2–88 years, with a mean age of 34.9 years (SD ±22.6) and a median age of 32 years (interquartile range 15–51 years). The number of individuals was equally distributed throughout the different age groups. The study population was of low income and educational level: 90% of adult individuals earned minimum Brazilian wages, and almost 80% had only elementary education or less. The primary drinking water source is the local brook (60% of the residences) and the domestic sewage receives no treatment.

**Table 1:** Selected socio-economic parameters and demographics of the study population in a rural community of Brejo do Amparo, Januária, Minas Gerais, Brazil.

Variables	Category	Total N (%)	Prevalence (%)	Parasite Load Eggs/g feces (Means ±SD) ^(d)^
**Sex** ^(a)^	Male	112 (48.9)	21.0	57.6±396.7
	Female	117 (51.1)	19.8	28.5±162.9
**Age Category** ^(a)^	≤ 10	38 (16.6)	7.3	8.9±35.1
	11–20	43 (18.8)	27.7	153.0±679.9
	21–40	61 (26.6)	27.9	36.0±91.8
	41–60	60 (26.2)	15.4	9.6±47.3
	>60	27 (11.8)	8.3	8.7±38.8
**Educational level** ^(b)^	No education	123 (59.4)		
	Primary school	33 (15.9)		
	Secondary school	43 (20.8)		
	Higher education	8 (3.9)		
**Income (Brazilian minimum wage)** ^(c)^	< 1 salary	19 (35.9)		
	1–2 salaries	16 (30.1)		
	> 2 salaries	18 (34.0)		
**Water Supply** ^(c)^	Covered well	21 (39.6)		
	Stream	32 (60.4)		
**Sewage system** ^(c)^	Rudimentary cesspool	47 (88.6)		
	Does not know or no answer	6 (11.4)		

^(a)^Variable evaluated by a questionnaire applied to each participant (n = 229)

^(b)^Variable evaluated by a questionnaire applied to each participant (n = 207). Children under 6 years of age were excluded from the analysis

^(c)^Variable evaluated by a questionnaire applied to the participants’ families (n = 53 residences).

(d) Parasite loads in males and females and in each age group are indicated as mean eggs per gram of feces ± standard deviation(SD), as determined by two KK slides from one fecal sample for each egg-positive individual.

**Fig 2:**
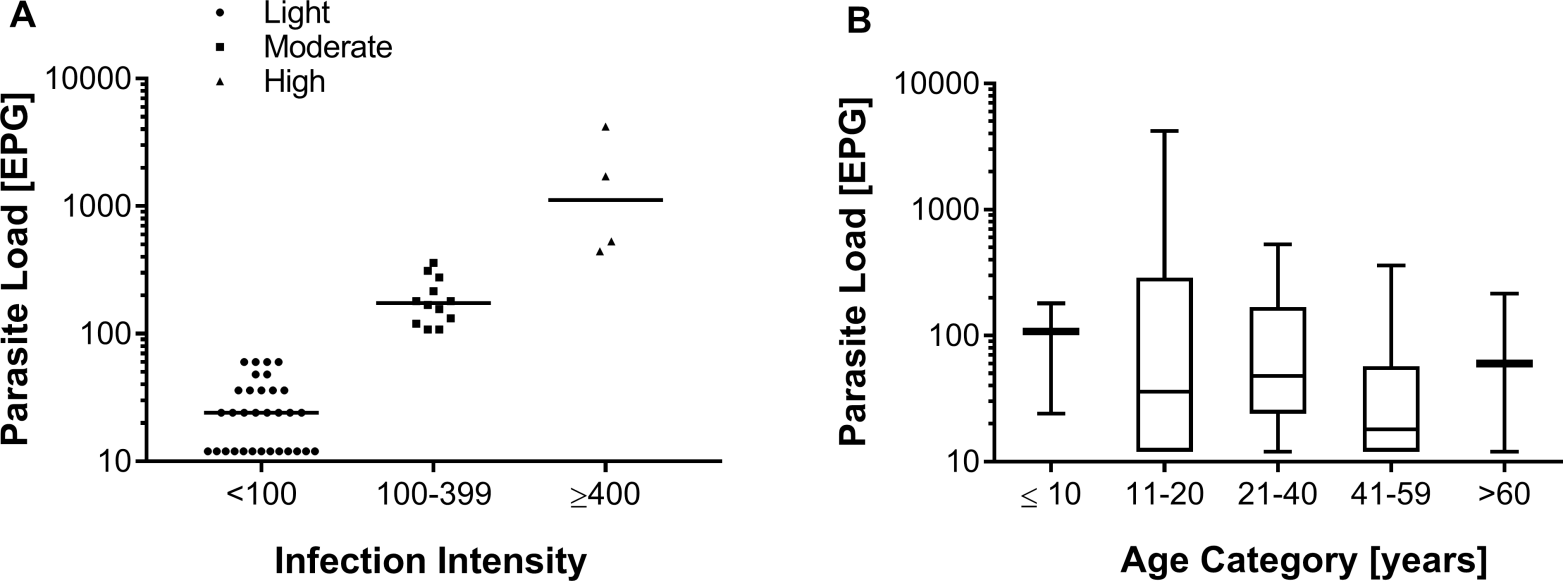
Classification of *Schistosoma mansoni*-infected individuals according to their parasitic load. **A:** Individual egg counts of one fecal sample analyzed with two Kato-Katz slides and classification of egg-positive individuals according to their parasite load. Infection intensity was determined by the number of eggs per gram of feces (EPG) and was classified as light (1–99 EPG, triangles), moderate (100–399 EPG, squares) or heavy (≥400 EPG, circles). Individual EPG values are plotted on a logarithmic scale and the horizontal bars indicate the mean EPG value in each category. **B:** Boxplots showing the median, interquartile ranges, and 95% intervals of the parasite load (EPG) by the different age groups and indicated on a logarithmic scale. Non-parametric Kruskal-Wallis test revealed no statistical significance between age groups (p > 0.05).

The initial fecal analyses performed with the saline gradient and the standard KK (two slides) methods revealed that 85 individuals were positive for protozoan cysts and 81 individuals eliminated helminth eggs in the fecal samples (Table 2). The most prevalent helminthic infections were intestinal schistosomiasis (20.4%) and hookworm (9.8%). The mean number of *S*. *mansoni* eggs in infected individuals was 210 ± 645.8 EPG. Among these 48 infected individuals, most (66.7%; n = 32) had a low parasite load of less than 100 EPG, 25% (n = 12) had a moderate infection, and 8.3% were heavily infected (Fig 2A). There was no statistically significant association between the prevalence and the intensity of schistosomiasis with gender. Also, the intensity of *S*. *mansoni* infection was similar among individuals of different age groups (Fig 2B).

**Table 2:** Prevalence of *Schistosoma mansoni* infection and other intestinal parasites in a rural community of the Municipality of Januária, Minas Gerais, Brazil.

	Number of infected	Prevalence (CI 95%)
**Intestinal protozoa (n = 249)**	**85**	34.1 (28.3–40.4)
***Entamoeba coli***	31	12.5 (8.6–17.2)
***Endolimax nana***	25	10.0 (6.6–14.5)
***Blastocystis hominis***	13	5.2 (2.8–8.8)
***Entamoeba histolytica/díspar***	9	3.6 (1.7–6.8)
***Giardia lamblia***	4	1.6 (0.4–4.1)
***Iodamoeba butschlii***	2	0.8 (0.1–2.9)
***Entamoeba hartmani***	1	0.4 (0.01–2.3)
	**Number of Infected**	**Prevalence (CI 95%)**
**Helminths (n = 235)**	81	34.5 (28.4–40.9)
Schistosoma mansoni*	48	20.4 (15.5–26.2)
**Hookworm**	23	9.8 (6.3–14.3)
***Enterobius vermicularis***	8	3.4 (1.5–6.6)
***Strongyloides stercoralis***	1	0.4 (0.01–2.3)
***Trichuris trichiura***	1	0.4 (0.01–2.3)
Coinfection (***S***. ***mansoni* + Protozoa)**	30	12.1 (8.6–16.7)
Coinfection (***S***. ***mansoni* + other helminths)**	11	4.7 (2.6–8.2)

* Positive for *Schistosoma mansoni*, as determined by two KK slides from one fecal sample.

### Comparison of different methods for the diagnosis of schistosomiasis

To evaluate the sensitivity of the technique recommended by the WHO (two KK slides from one fecal sample) and other parasitological tests to identify *S*. *mansoni* infection, we performed thorough parasitological examinations using three fecal samples. Moreover, urine samples collected from the participating individuals were tested for the circulating cathodic schistosome antigen using the rapid urine test (POC-CCA), as described above.

**Fig 3:**
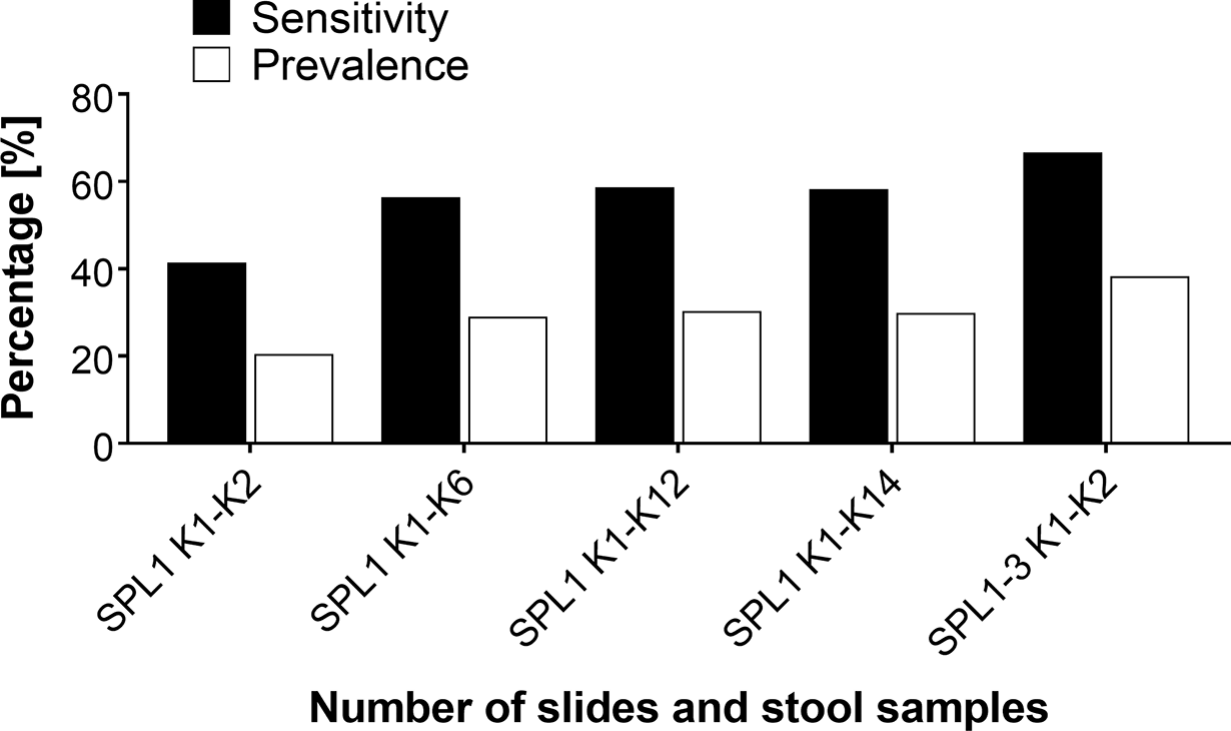
Prevalence of intestinal schistosomiasis and sensitivity of the Kato-Katz method, according to the number of examined slides and stool samples. Prevalence of *Schistosoma mansoni* infection (white bars), as determined by the analysis of one fecal sample with two (SPL1 K1-K2), six (SPL1 K1-K6), 12 (SPL1 K1-K12) or 14 slides (SPL1 K1-K14), or obtained with the analysis of two slides prepared from each of three fecal samples (SPL1-3 K1-K2). The sensitivity of the different numbers of Kato-Katz slides examined (black bars) was calculated in relation to the reference standard, which included the combined results of 18 Kato-Katz slides, the saline gradient, and the Helmintex methods.

The inclusion of additional parasitological methods for schistosome diagnosis resulted in the detection of a much higher number of infected individuals within the study population (Table 3). The apparent prevalence rose from 20.4 to 29.9%, when the number of KK slides was increased from two to 14 slides, or to 38.3%, when we used two slides prepared from each of the three fecal samples. Other parasitological methods that used a higher amount of fecal matter, such as the saline gradient and Helmintex, also detected a higher number of *S*. *mansoni*-infected individuals (Table 3). Overall, and taking into consideration the results of all the parasitological methods (reference standard), the prevalence of intestinal schistosomiasis reached 45.9%, which represents a 2.3 times increase in relation to the WHO’s recommended standard KK procedure. The reference standard was used to evaluate the efficacy of each of the diagnostic methods tested. For the fecal techniques, the best performance was obtained with the modified Helmintex method, which identified schistosome eggs in feces of 88 individuals (40.4% of prevalence). This parasitological method showed a high sensitivity (86.6%) and the highest degree of concordance in relation to the reference standard (kappa = 0.84) (Table 3). The analysis also demonstrated that the sensitivity of the KK method increased from 41.4% with two slides from one fecal sample to up to 66.7% with six slides from three fecal samples. In comparison, if only one fecal sample was processed, the sensitivity remained around 60%, even when the number of examined slides was increased to 12 or 14 (Table 3 and Fig 3). The improved performance of the KK method due to an increased number of examined slides (14 slides) or increased sampling effort (three fecal samples) is shown in Fig 3.

**Table 3:** Performance of different parasitological and immunochromatographic methods for the detection of intestinal schistosomiasis in comparison with the reference standard (18 Kato-Katz slides, saline gradient, and Helmintex).

Method	Prevalence (%)	Sensitivity % (CI 95%)	Kappa Index (CI 95%)
**SPL1 K1-K2**	20.4	41.4 (32.8–50.5)	0.42 (0.31–0.52)
**SLP1 K1-K6**	29.0	56.4 (47.0–65.3)	0.56 (0.44–0.67)
**SLP1 K1-K12**	30.3	58.7 (49.3–67.5)	0.58 (0.46–0.70)
**SLP1 K1-K14**	29.9	58.2 (48.8–67.0)	0.58 (0.46–0.70)
**SLP1-3 K1-K2**	38.3	66.7 (57.3–74.9)	0.63 (0.50–0.76)
**Saline Gradient**	21.3	44.7 (35.0–54.7)	0.46 (0.34–0.58)
**Helmintex**	40.4	83.8 (75.6–89.6)	0.84 (0.71–0.97)
**POC-CCA**	47.4	64.9 (55.6–73.1)	0.34 (0.22–0.47)

Data shows the prevalence, sensitivity, and kappa index of concordance for the Kato-Katz technique obtained with the analysis of one fecal sample using two (SPL1 K1-K2), six (SPL1 K1-K6), 12 (SPL1 K1-K12), and 14 slides (SPL1 K1-K14), or obtained from two slides prepared from each of three fecal samples (SPL1-3 K1-K2), or obtained with the saline gradient, Helmintex, or with POC-CCA methods.

**Fig 4:**
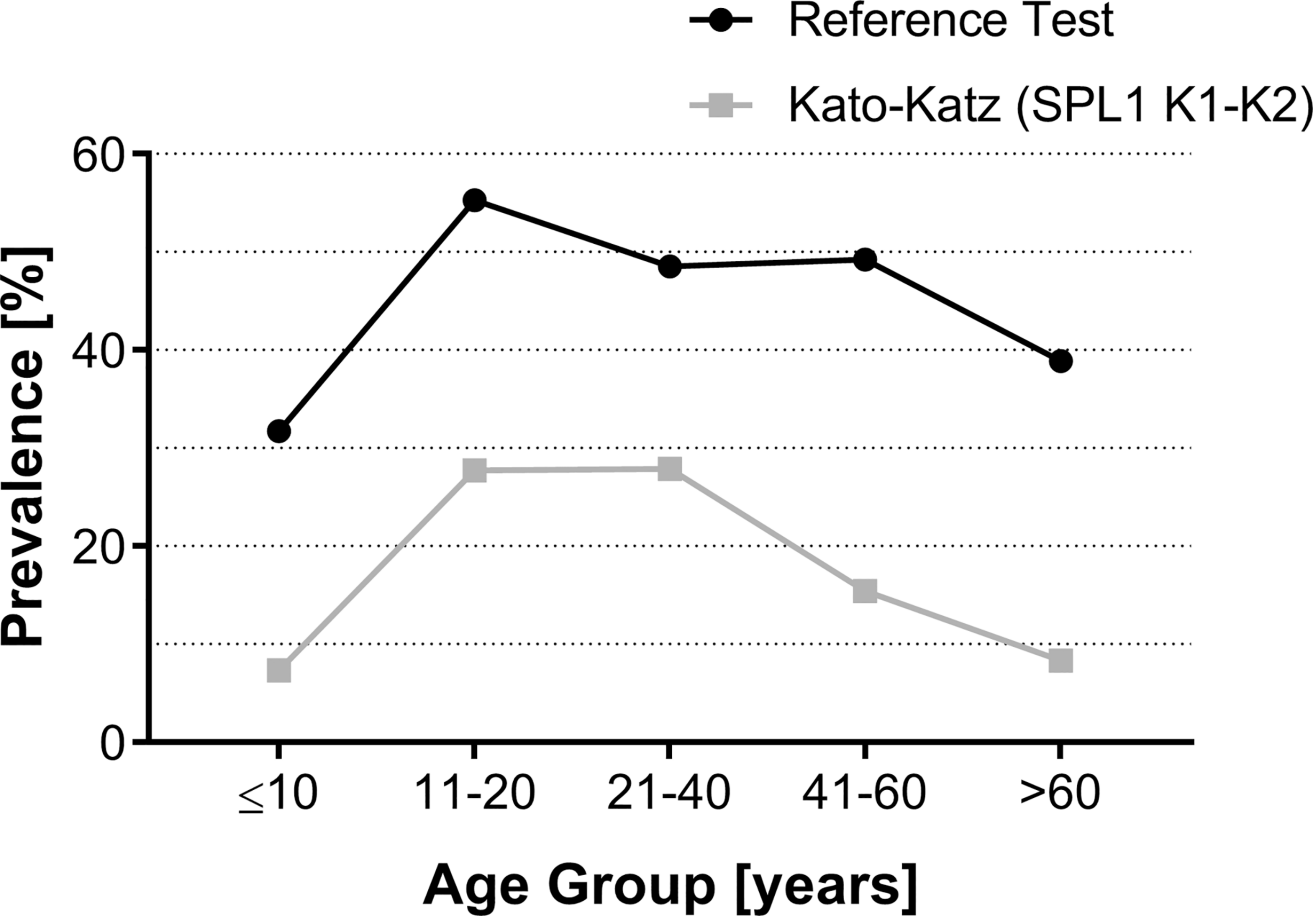
Prevalence profile of intestinal schistosomiasis in an endemic population divided by different age groups according to the different parasitological methods. Black circles indicate the prevalence profile in the population considering the sum of all parasitological methods used (reference standard: 18 Kato-Katz slides, saline gradient, and Helmintex); grey squares indicate the prevalence profile considering the recommended two KK slides from one fecal sample. Prevalence values (%) for each age group are indicated.

Fig 4 shows the prevalence of *S*. *mansoni* infection for the different age groups and as a function of the parasitological methods, e.g. the standard KK method (2 slides from one fecal sample) versus the reference standard **(**18 Kato-Katz slides, saline gradient, and Helmintex). Using the reference standard, we found that children and young adults (11–20 years of age) had the highest prevalence (55%) for *S*. *mansoni* infection. In contrast, the prevalence was reduced to less than 50% in the other age groups, being further reduced in the elderly (older than 60 years of age). Importantly, the prevalence found in each age group, considering the combination of all parasitological exams (reference standard), was 1.7 to 4.7 times higher than the prevalence obtained with the recommended two KK slides from one fecal sample (Fig 4).

### Classification of infected individuals and performance of the different parasitological methods according to the parasite load

The parasite load in *S*. *mansoni* infected individuals was determined by counting the eggs found in two KK slides from one fecal sample and converting the counts in eggs per gram of feces (EPG), according to standard procedures recommended by the WHO [14]. We assigned an EPG value of 1 for the individuals who were not detected by two KK slides, but who were found positive when additional slides were analysed or when other fecal exams were used. Thus, we classified 102 individuals with a light parasite load (EPG: 1–99), 12 individuals with a moderate parasite load (EPG: 100–399), and four individuals with a heavy parasite load (EPG: 400 or more). Therefore, most of the infected individuals within the studied population had a light parasitic infection.

Next, we analyzed the performance of the parasitological methods in relation to the parasite load, with the individuals with a light infection being arbitrarily divided into three subgroups (Table 4). All the diagnostic methods readily detected individuals with heavy to moderate infections. On the other hand, the diagnostic methods decreased their sensitivity to detect individuals with a low parasite load, especially in fecal samples with less than 12 EPG. In this case, the best performance of the KK method (SPL1-3 K1-K2) reached a sensitivity of only 40%. In the group with a very low parasite load, the saline gradient and the rapid urine test had sensitivities of 33.9 and 50.8%, respectively. The Helmintex method showed the highest sensitivity for the group with very low parasite load (84.1%).

**Table 4:** Sensitivity of different diagnostic methods for the detection of intestinal schistosomiasis considering the parasite load, as defined by egg counts of two Kato-Katz slides.

Classification by Parasite Load (EPG value) Sensitivity (%) of each diagnostic method					
Diagnostic Method	Heavy (EPG > 399) % Sensitivity	Moderate (EPG: 100–399) % Sensitivity	Light		
			(EPG: 99–50) % Sensitivity	(EPG: 49–12) % Sensitivity	(EPG < 12) % Sensitivity
SPL1 K1-K2	100 (04/04)	100 (12/12)	100 (04/04)	100 (28/28)	0 (0/70)
SPL1 K1-K6	100 (04/04)	100 (12/12)	100 (04/04)	100 (28/28)	22.6 (14/62)
SPL1 K1-K12	100 (04/04)	100 (11/11)	100 (04/04)	100 (28/28)	27.4 (17/62)
SPL1 K1-K14	100 (04/04)	100 (11/11)	100 (04/04)	100 (28/28)	27.0 (17/63)
SPL1-3 K1-K2	100 (04/04)	100 (12/12)	100 (04/04)	100 (28/28)	40.0 (24/60)
Saline Gradient	100 (04/04)	100 (06/06)	33.3 (01/03)	50.0 (11/22)	33.9 (20/59)
Helmintex	100 (03/03)	100 (11/11)	75.0 (03/04)	75.0 (18/24)	84.1 (53/63)
POC-CCA	100 (04/04)	100 (10/10)	100 (04/04)	77.8 (21/27)	50.8 (34/67)

Data show the sensitivity (%) of each diagnostic method and the number of individuals detected positive for intestinal schistosomiasis versus the total number of examined individuals (in brackets), according to parasite load classification. Individuals with a light infection were arbitrarily divided into three subgroups with egg counts of 99–50 eggs per gram of feces (EPG), 49–12 EPG, and less than 12 EPG.

### Performance of the POC-CCA as diagnostic method for intestinal schistosomiasis

The POC-CCA identified 108 out of a total of 228 individuals as infected, which resulted in a prevalence of 47.4% and a sensitivity of 64.9%, when compared with the reference standard **(**18 Kato-Katz slides, saline gradient, and Helmintex). The sensitivity of the POC-CCA was superior to the saline gradient and comparable to the results obtained with six KK slides from three fecal samples. However, the kappa index of the urine test was considerably lower than that obtained with the other parasitological tests (Table 5).

**Table 5:** Performance of the rapid urine test for circulating cathodic antigen of *Schistosoma mansoni* (POC-CCA), as compared with the reference standard of a positive result in any of the used parasitological methods.

Method	TP	FP	TN	FN	Prevalence % (n positives/ n total)	Sensitivity % (CI 95%)	Specificity % (CI 95%)	PPV (%)	NPV (%)	Kappa (CI 95%)
POC-CCA *	73	35	81	39	47.4 (n = 108 of 228)	64.9 (55.6–73.1)	69.2 (60.4–76.9)	66.7 (57.3–74.9)	67.5 (58.7–75.2)	0.34 (0.22–0.47)
POC-CCA**	30	2	114	82	14.0 (n = 32 of 228)	26.8 (19.5–35.7)	98.3 (93.9–99.5)	93.8 (79.9–98.3)	58.2 (51.2–64.9)	0.25 (0.17–0.35)

The POC-CCA test was evaluated considering trace results as a positive result (*), as indicated by the manufacturer, or considering trace results as a negative result (**). The data show the number of true positive (TP), false positive (FP), true negative (TN), and false negative (FN) individuals, as well as the prevalence (%), sensitivity, specificity, and kappa index, together with the respective confidence intervals (CI), of concordance of each test.

**Fig 5:**
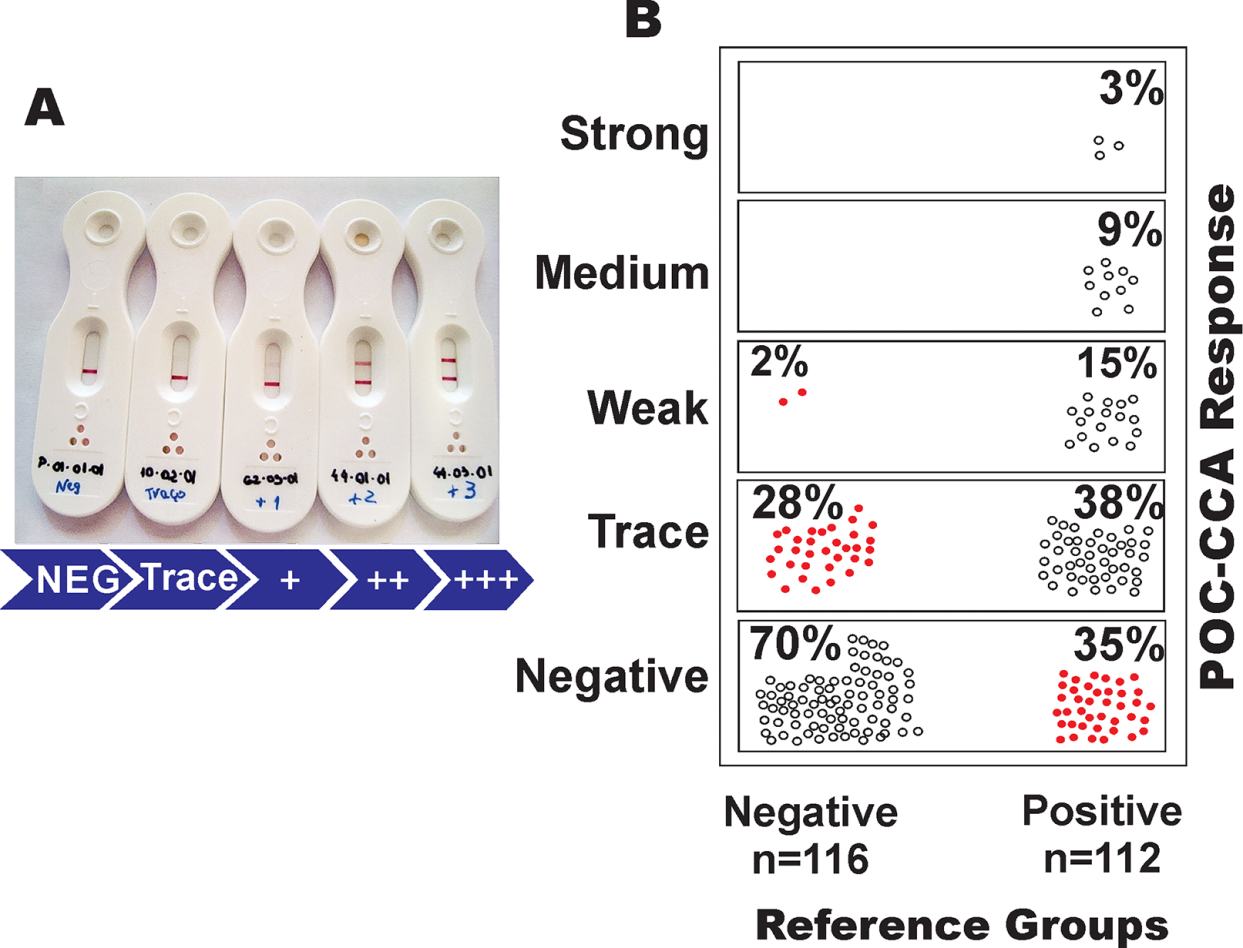
Performance of the rapid urine test (POC-CCA) for the diagnosis of *Schistosoma mansoni* infection. **A:** Photograph showing the different reactions possible with the POC-CCA: negative, trace, weak (+), medium (++) and strong (+++). **B:** Distribution of the POC-CCA results in individuals from an endemic area classified as negative (n = 116) or positive (n = 112) for *S*. *mansoni* infection by extensive parasitological testing (Reference standard: 18 Kato-Katz slides, saline gradient, and Helmintex); total n = 228). Data indicate the percentages of POC-CCA reactivities in each group of parasitologically negative or positive individuals. Red circles indicate discordant results in comparison with the reference standard (false positive: 28 and 2%; or false negative: 35%).

The performance of the POC-CCA is illustrated in Fig 5. The visual scores ranged from negative to trace, weak (+), moderate (++), and strongly positive (+++), with trace results considered a positive reaction for *S*. *mansoni* infection, as recommended by the manufacturer (Fig 5A). Comparing the POC-CCA result with the other parasitological analyses, we observed that, of the 139 negative individuals in the parasitological tests, only 116 participants provided urine samples for the POC-CCA test. Of these individuals, 81 (70%) were also found not reactive (negative) in the urine test. However, 33 urine samples (28%) from the individuals found negative by the other parasitological tests showed a trace reaction and another two samples (1.7%) of parasitologically negative individuals had a weak positive result (+) (Fig 5B). Among these 35 individuals, only four (11.4%) individuals had a hookworm infection and eight (22.9%) individuals presented with protozoan cysts in their feces.

From 118 individuals found positive for *S*. *mansoni* eggs in any of the parasitological exams (reference standard), 112 participants provided urine samples. Out of these 112 samples, 73 (65%) were tested positive for the circulating cathodic antigen of *S*. *mansoni* and were in agreement with the results of the other parasitological exams. The results were classified as trace, weak (+), medium (++), or strongly positive (+++) in 43 (38%), 17 (15%), 10 (9%), and three (3%) of the examined urine samples, respectively (Fig 5B). In contrast, 39 urine samples (35%) from egg-positive individuals were not reactive in the urine test and, therefore, were misclassified as uninfected (false negatives). Interestingly, the mean EPG value from these missclassified individuals was considered as very low (mean EPG: 4.3; minimum: 1 EPG, maximum: 36 EPG).

Out of the 73 samples that were positive according to the reference standard and in the POC-CCA, 59 (81%), 10 (14%), and four (5%) individuals were considered to have a light, moderate or high parasite load, respectively. A significantly positive correlation was found between the scores of the POC-CCA and intensity of infection, as determined by individual EPG values (R = 0.537; *p* = 0.0001). The agreement between POC-CCA and the reference standard, as the sum of all parasitological exams, showed a low concordance (κ = 0.34), which was even lower when trace results in the urine test were considered as a negative result (κ = 0.25) (see also Table 5).

### Combination of methods for an improved diagnosis of intestinal schistosomiasis

Since the KK method is the recommended technique for the diagnosis of intestinal schistosomiasis [14], we compared the combination of more KK slides and fecal samples with the modified Helmintex method, saline gradient, and the rapid urine test (POC-CCA). The combination of two KK slides from one fecal sample (1SPL K1-K2) with Helmintex or of six slides from three fecal samples (SPL1-3 K1-K2) with POC-CCA resulted in the highest prevalence rates (45.4% and 60.3%) and highest sensitivity rates (90.0% and 88.3%) (Table 6). Looking for easily applicable diagnostic methods with improved sensitivity for epidemiological studies, we found that the combination of the KK method with the POC-CCA test produced better results when the number of slides and fecal samples was increased (Table 6).

**Table 6:** Prevalence, sensitivity and specificity of different diagnostic tests when combined with the Kato-Katz method, as compared with the reference standard (18 Kato-Katz slides, saline gradient, and Helmintex).

Combination of Diagnostic Methods	Prevalence (%)	Combined sensitivity parallel tests (%)	Combined specificity parallel tests (%)
**SPL1 K1-K2 + GRAD**	27.8	67.6	100
**SPL1 K1-K6 + GRAD**	31.3	75.9	100
**SPL1-3 K1-K2 + GRAD**	40.1	81.6	100
**SPL1 K1-K2 + HTX**	45.4	90.0	100
**SPL1 K1-K6 + HTX**	48.7	92.5	100
**SPL1-3 K1-K2 + HTX**	56.9	94.3	100
SPL1 K1-K2 + POC-CCA*	48.4	79.4	69.3
SPL1 K1-K2 + POC-CCA**	33.0	57.1	98.3
SPL1 K1-K6 + POC-CCA*	54.1	84.7	69.3
SPL1 K1-K6 + POC-CCA**	39.3	68.1	98.3
SPL1-3 K1-K2 + POC-CCA*	60.3	88.3	69.3
SPL1-3 K1-K2 + POC-CCA**	46.6	75.6	98.3

The Kato-Katz method with an increasing number of slides or fecal samples was combined with the saline gradient (GRAD), Helmintex (HTX) and the rapid urine test (POC-CCA). Rapid urine test (POC-CCA) with trace results considered positive(*) and trace results considered negative(**).

## Discussion

Human schistosomiasis is still considered a parasitic infection with global health impact as it affects about 250 million people in 78 countries and more than 700 million individuals are estimated to live at risk of infection [3,4,16]. In the Americas, the only schistosome species is *S*. *mansoni* and recent estimates indicated about 1.8 million infected individuals and approximately 25 million people living at risk of infection, with most of the cases occurring in Brazil [9]. In Brazil, transmission of schistosomiasis occurs in 19 states with a larger presence in the northeastern states as well as Minas Gerais, and Espírito Santo [41]. Recent data released by the Brazilian Ministry of Health in 2012 indicated a positivity rate of 4.5% of examined individuals residing in the endemic areas covered by the National Program for Schistosomiasis Control [42].

The large-scale parasitological screening, individual diagnosis, and ongoing treatment with praziquantel promoted by the NPSC led to a considerable reduction of infection rates, severe clinical cases, and transmission of *S*. *mansoni* in endemic areas [21, 42]. For field-based diagnosis, the recommended tool for the detection of intestinal schistosomiasis is the KK thick-smear method [13], which detects schistosome eggs in two KK slides from one fecal sample [14]. However, it has been shown, in different endemic settings, that this technique is not sensitive enough to detect schistosomiasis in individuals with low parasite burden [17,25,43–47], being even less sensitive when only one KK slide was examined, as usually occurs during the interventions promoted by the NPSC [12,19,20]. Thus, the Brazilian Ministry of Health recommends the examination of a higher number of KK slides in areas where the parasite burden is supposed to be low [42].

In the present study, we tested the sensitivity of an increasing number of KK slides using up to three fecal samples and compared its performance with other parasitological methods in an area endemic for intestinal schistosomiasis. By performing thorough parasitological exams, we aimed to get close to the ‘real’ picture of *S*. *mansoni* infections in this endemic region, which is located within the area of action of the NSPC, but had not suffered any intervention in the two years before the beginning of the present study. The diagnostic effort presented herein allowed us to evaluate different parasitological methods in the light of a strong reference standard, uniquely defined in this study.

Most of the population from the rural area studied herein had no adequate water supply and sanitation. While waterborne protozoan infections were common, other intestinal helminth infections were less frequent. *S*. *mansoni* infection was initially estimated to be 20.4%, after examination of two KK slides from one fecal sample, which led the area to be classified as with moderate risk of infection [48]. The classification of infected individuals according to their parasite load [14] confirmed that two thirds of the initially diagnosed individuals harbored a light *S*. *mansoni* infection and less than 10% had a heavy infection. After performing additional KK slides and other parasitological methods, the prevalence rose to 45.9%, which represented a 2.3 times increase when compared with the initial exams and indicated nearly half of the examined population as infected. As revealed by previous studies in areas of low transmission of *S*. *mansoni* [12,19,20,25], the prevalence of infection for this parasite in the area studied herein was largely underestimated when only the standard KK method was used. The prevalence profile for intestinal schistosomiasis in different age groups revealed herein matched that from other studies [4, 49,50]. However, if the standard method of two KK slides was compared with our reference standard (18 KK slides + saline gradient + Helmintex), we identified up to 4.7 times higher prevalences in the different age groups. This is in line with results published previously [19, 20], showing that an increase in the number of examined KK slides considerably augmented the number of egg-positive individuals. However, and this goes beyond the already existing data on the evaluation and performance of multiple KK slides, we showed that even using the superior version of the KK technique, which involves analyzing two slides from three different fecal samples, we still missed more than one third of the infected population.

Besides the KK technique, the other parasitological tests composing our reference standard included a saline gradient using 500 mg of fecal matter from the first fecal sample [23] and performed modified Helmintex method [38], which used up to 30 grams of feces. Using these methods, a considerable number of additional egg-positive individuals were detected, with the Helmintex method presenting the best performance and a sensitivity of over 80%. Initially, the Helmintex method was described of being 30 times more sensitive than the standard KK method [24], which is mainly due to the high amount of examined fecal matter, the successive sieving and concentration processes and the separation and distinction of eggs by paramagnetic beads and additional staining methods in the modified version [38]. A study investigating a low transmission area in the northeast of Brazil using the Helmintex method showed similar results to ours published [51]. However, it has to be emphasized that, in the present setting, none of the parasitological methods tested herein was able to detect eggs in every positive sample. In this context it is interesting to note, that in seeding experiments with 30 grams of feces, the recovery of schistosome eggs in fecal samples processed by the Helmintex method was about 27%, only [38]. Further, it was not the aim of the study to evaluate and compare the different methods in terms of applicability in field surveys, as well as operational, personnel, and logistics and other factors that influence their implementation, as stated by others [19, 52–54].

An interesting alternative to the time consuming and labor intensive parasitological methods are rapid immunochromatographic tests for circulating antigens. Therefore, we included the commercialized rapid urine test (POC-CCA) [28] in our study and evaluated it in comparison with our parasitological reference standard The POC-CCA has shown promising results for the detection of intestinal schistosomiasis in various settings in Africa and Asia [32–34, 55,56]. When the *S*. *mansoni* egg-positive individuals tested herein were classified according to their parasite load, the parasitological tests and the POC-CCA readily detected the individuals with heavy or moderate infections. In contrast, all tests (parasitological and the POC-CCA) showed reduced sensitivities when individuals with a low (99–12 EPG) or very low (less than 12 EPG) parasite load had to be detected. Especially in the case of very low parasite load, the KK technique, at its best, only detected 40% of the infected individuals. In the case of the individuals with very low parasite load, the POC-CCA and the Helmintex methods showed the best performance with sensitivities of more than 50 and 84%, respectively.

The rapid urine test (POC-CCA) has been successfully tested in different regions of Africa and Asia [32,34, 56–58] and there are initiatives which favor this test for screening and mapping of intestinal schistosomiasis and improve transmission control and the elimination of schistosomiasis [59,60]. However, the epidemiological situation of intestinal schistosomiasis in most areas in Brazil is different from that found in many endemic settings in other tropical countries. This is probably because the country has a national program for schistosomiasis control since the 1970s with regular intervals of diagnosis and treatment rounds in endemic populations. Data from the Brazilian Ministry of Health [42] and risk mapping of schistosomiasis in the country [61–63] indicated a considerable decrease in infection rates and high prevalence risk areas with ongoing interventions [42, 63]. However, these claims might be overly optimistic since they are based on data from one KK slide from one fecal sample. In any case, according to the government-published data, as a result of the NPSC interventions, the parasite burden, significant morbidity, and mortality rates decreased during the last two decades [42]. We evaluated the performance of POC-CCA and compared it with the reference standard to detect *S*. *mansoni* infection in individuals of a community where NPSC’s interventions including varying rounds of treatment had been promoted. The POC-CCA showed a sensitivity of approximately 65%, which is superior to that obtained with the saline gradient method, comparable with the results obtained with KK variant using six KK slides from three fecal samples, and inferior to the sensitivity found with Helmintex, when the criteria of evaluation were used as indicated by the manufacturer that is, if ‘trace’ was considered a positive result. A similar result for the sensitivity of POC-CCA and comparison with the performance of multiple KK smears was obtained in an endemic area in Africa [64]. In our study, the main shortcoming of the rapid urine test (POC-CCA) was a low concordance with the reference standard, since we found 30 and 35% of false positive and false negative results, respectively. This low concordance for the rapid urine test was not observed in other studies where parasitological efforts for detection of schistosome eggs in feces were far less rigorous [35, 65]. Also, the discrepancy might be partially explained by the discontinuous distribution of eggs in the fecal matter, intermittent egg excretion, a small number of female worms, or by occult infections with just one sex or aging worms. This is somewhat expected in elderly individuals since they rarely visit contaminated water streams and are, therefore, less prone to reinfection [32,43,46,66,67]. If ‘trace’ was not considered as positive, then the specificity increased to more than 98%, but the sensitivity dropped to less than 27%, which we consider insufficient for a screening method. In a recent study, the performance of POC-CCA was compared with that of a KK test with two slides of one fecal sample, as recommended by WHO, and without further extensive parasitological testing [65]. Even in that experimental setting, without a strong reference standard, the rapid urine test had a considerable percentage of false positive results, and that occurred even for individuals from an area considered as non-endemic for schistosomiasis. Additionally, 14% were classified as negative by the urine test, but these were proven to be positive during parasitological exams [65]. Previous studies have reported cross-reactivity between schistosomes and other intestinal helminths or other clinical conditions that can lead to a false positive POC-CCA result [68–70]. However, we were not able to correlate any intestinal protozoan or helminth infection with a ‘trace’ or positive POC-CCA result.

In order to improve the performance of POC-CCA test and elucidate the situation of individuals who were tested as ‘trace’, prior concentration of urine by lyophilization significantly improved the concordance of the test in individuals with low parasite burden [70]. Also, recent investigations on the specificity and sensitivity of methods for the detection of circulating anodic antigen (CAA) from schistosomes seem to be even more promising [71–74].

To reach a maximum sensitivity and specificity and indicate alternatives for schistosomiasis control programs, we tried to combine the standard KK method and different modifications of this technique with the other parasitological methods or the POC-CCA. The best KK variant tested herein (six slides prepared from three fecal samples) achieved a sensitivity of 82, 88, and 94% when combined with the saline gradient, POC-CCA or Helmintex methods, respectively. Whether any of these scenarios is applicable to large-scale national control programs has to be carefully evaluated, considering logistic and economic aspects [54]. In any case, maybe a first parasitological test has to be combined with a second more specific test for schistosomiasis in order to join efforts against soil-transmitted helminthiasis and schistosomiasis [54,59,75]. Further, we believe that in areas of low endemicity or low intensity infections, serology or molecular biology, as proposed elsewhere [11,25,76–78], might be valuable alternatives to be included as additional diagnostic procedures. We are currently investigating the performance of molecular biological methods and serology in the parasitologically well-defined population studied herein.

In conclusion, we showed that in endemic areas of intestinal schistosomiasis with low-intensity infections, the actual prevalence can be underestimated by up to 4.7 times when measured by the recommended standard procedure. The rigorous parasitological testing of three fecal samples allowed us to evaluate parasitological and immunochromatographic methods for diagnosis of infection with *S*. *mansoni*. The KK technique, even at its best was able to detect only two-thirds of the infected individuals. The best sensitivity rate (over 80%) was achieved with the Helmintex method. However, in its present form, Helmintex is not applicable for large-scale screening due to the required sample size and the time-consuming sieving and sedimentation processes [38], but might be an adequate reference standard or gold standard for the evaluation of newly developed, field-based diagnostic tools. In addition, the performance of the POC-CCA was in the range of the best KK variant (six slides from three fecal samples), but a high number of individuals were not correctly diagnosed (false positive or false negative). Furthermore, studies are underway, in order to re-evaluate the use of standard serological methods and PCR-based detection of parasite DNA with our well-defined biological samples. We believe that a combination of methods has to be implemented since the schistosomiasis control programs in different regions of the world are moving from morbidity control towards transmission control and elimination.

## Supporting information

S1 Fig Geographical localization of Minas Gerais State within Brazil (small red window) and localization of the endemic area in the Municipality of Januária, northern region of Minas Gerais (zoom). Source: https://pt.wikipedia.org/wiki/Janu%C3%A1ria#/media/File:MinasGerais_Municip_Januaria.svg.
(TIF)
