# Supplementary figures and images for: Evaluation of diagnostic methods for the detection of intestinal schistosomiasis in endemic areas with low parasite loads: Saline gradient, Helmintex, Kato-Katz and rapid urine test
Source: PLoS Negl Trop Dis. 2018 Feb 22;12(2):e0006232. doi: 10.1371/journal.pntd.0006232 (PMC5823366; doi:10.1371/journal.pntd.0006232)

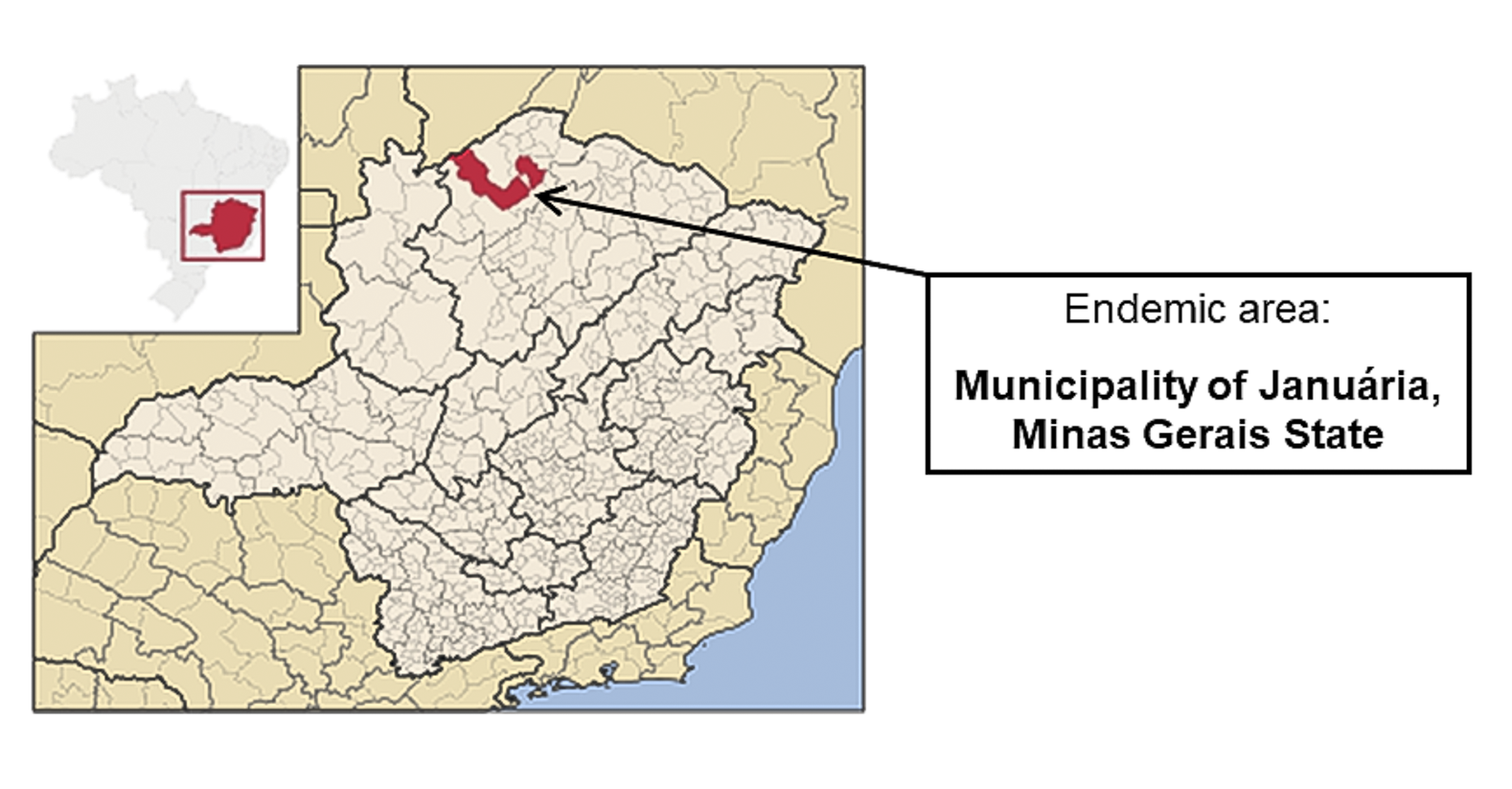

Supplement: S1 Fig — Source: https://pt.wikipedia.org/wiki/Janu%C3%A1ria#/media/File:MinasGerais_Municip_Januaria.svg. (TIF) [file pntd.0006232.s001.tif]
